# Development and Evaluation of Paclitaxel‐Loaded Self‐Nanoemulsifying Drug Delivery System for Enhanced Cancer Therapy: A Promising Alternative to Cremophor EL–Based Formulation

**DOI:** 10.1049/nbt2/4890307

**Published:** 2026-05-22

**Authors:** Alshaimaa M. Almehmady, Amerh A. Alahmadi, Abrar Hakami, Ehab M. M. Ali, Abdulaziz A. Kalantan, Amjad Aljagthmi, Tarek A. Ahmed

**Affiliations:** ^1^ Department of Pharmaceutics, Faculty of Pharmacy, King Abdulaziz University, Jeddah, 21589, Saudi Arabia, kau.edu.sa; ^2^ Department of Biochemistry, Faculty of Science, King Abdulaziz University, Jeddah, 21589, Saudi Arabia, kau.edu.sa; ^3^ Research and Innovation, King Faisal Specialist Hospital and Research Center, Riyadh, Saudi Arabia, kfshrc.edu.sa

**Keywords:** breast cancer, drug delivery systems, nanoemulsion, paclitaxel, pharmacodynamics

## Abstract

**Background and Purpose:**

Paclitaxel’s clinical use is limited by poor aqueous solubility and Cremophor EL–related toxicity in commercial formulations. This study aimed to develop a self‐nanoemulsifying drug delivery system (SNEDDS) to improve paclitaxel solubility, bioavailability, and anticancer efficacy.

**Experimental Approach:**

Paclitaxel‐loaded SNEDDSs were prepared using oleic acid, Tween 80, and polyethylene glycol (PEG) 400 in different ratios and characterized for particle size, polydispersity index (PDI), zeta potential, and solubility. The optimized formulation (F1) was assessed for cytotoxicity, cell cycle distribution, apoptosis, mitochondrial membrane potential (MMP), and nuclear morphology in MCF‐7 breast cancer cells.

**Key Results:**

Formulation F1 (10% oleic acid, 10% PEG 400, and 80% Tween 80) exhibited the highest solubility, smallest particle size, and lowest PDI, with near‐neutral zeta potential ensuring stability. F1 demonstrated superior cytotoxic activity, inducing G_2_/M arrest (41.8%) and total apoptosis of 70.6%, mainly in the early phase (64.4%), compared to pure paclitaxel and Paxol. MMP and 4^′^, 6‐diamidino‐2‐phenylindole (DAPI) assays confirmed mitochondrial‐mediated apoptosis and nuclear fragmentation, consistent with paclitaxel’s mechanism of microtubule stabilization and mitotic catastrophe.

**Conclusion and Implications:**

Encapsulation of paclitaxel into SNEDDS significantly enhanced solubility, cellular uptake, and proapoptotic activity. The optimized F1 formulation provides a promising nanocarrier platform for improving paclitaxel’s therapeutic performance and may serve as a safer, more effective alternative to Cremophor EL–based products for breast cancer treatment.

## 1. Introduction

Paclitaxel is a broad‐spectrum chemotherapeutic agent widely used in the management of several solid tumors, including breast, ovarian, and lung cancers [1]. Since its clinical approval in 1994, paclitaxel has become integral to frontline cancer therapy regimens owing to its potent antiproliferative activity [2]. Paclitaxel exerts its effect by binding to the β‐subunit of tubulin, promoting microtubule polymerization and stabilization while preventing depolymerization. This disrupts essential microtubule dynamics required for mitosis, causing G_2_/M phase arrest and triggering apoptotic cell death [3, 4]. Currently, paclitaxel has been used for two decades as a single agent and in combination chemotherapy due to its synergistic effects and ability to overcome multidrug resistance in specific cancer types [5].

Despite its clinical success, paclitaxel’s utility is significantly hindered by its extremely poor aqueous solubility (<0.01 mg/mL) and high molecular weight (MW ~853.9 Da) [6]. The commercial formulation Taxol solubilizes paclitaxel in Cremophor EL and ethanol; however, this delivery strategy presents several limitations, including instability upon dilution with aqueous media and risk of precipitation due to concentrations exceeding intrinsic solubility [7]. More critically, Cremophor EL is associated with severe adverse effects such as hypersensitivity reactions, neurotoxicity, nephrotoxicity, and metabolic disturbances [7]. These toxicities necessitate steroid and antihistamine premedication, complicating treatment and reducing patient compliance [8]. Additionally, dose‐limiting toxicities such as neutropenia and peripheral neuropathy restrict optimal clinical dosing and compromise therapeutic outcomes [9].

To overcome these limitations, nanotechnology‐enabled delivery systems have emerged as promising strategies to enhance paclitaxel solubilization, bioavailability, and safety. Several nanobased paclitaxel formulations—such as nanoparticle albumin‐bound paclitaxel (Abraxane), polymeric micelles, liposomal systems, and polymer–drug conjugates—have demonstrated improved tolerability and clinical activity [10–14]. Nonetheless, challenges including complex manufacturing, high cost, limited drug‐loading capability, and stability issues remain barriers to widespread adoption.

Self‐nanoemulsifying drug delivery systems (SNEDDS) represent an attractive alternative due to their simplicity, scalability, and ability to spontaneously form nanoemulsions in physiological fluids [15]. SNEDDS are composed of oils, surfactants, and cosurfactants/cosolvents that generate nanosized droplets upon dispersion, markedly enhancing surface area for drug dissolution and absorption [16]. These systems improve the oral bioavailability of poorly water‐soluble drugs, maintain them in a solubilized form, and have demonstrated success in enhancing the delivery of anticancer agents such as docetaxel, mesalamine, and tamoxifen [17]. Although paclitaxel‐loaded SNEDDS and related self‐emulsifying systems have been previously investigated, most reported studies were primarily designed to improve absorption and bioavailability, often through more complex approaches such as coadministration with P‐glycoprotein inhibitors [18], phospholipid‐drug complexes, emulsion systems, micelles, and nanosuspensions [19]. Accordingly, there is a high demand for developing a simpler and pharmaceutically practical liquid SNEDDS that enhances the in vitro anticancer efficacy of paclitaxel and serves as a potential alternative to Cremophor EL–based formulations.

This study aimed to develop and characterize a paclitaxel‐loaded SNEDDS using oleic acid, Tween 80, and polyethylene glycol (PEG) 400 to enhance the drug’s solubility and anticancer efficacy. The promising formulation was further evaluated for its in vitro cytotoxic, apoptotic, and cell cycle–modulating effects in MCF‐7 breast cancer cells, along with mechanistic investigations including mitochondrial membrane potential (MMP) and nuclear morphology assessments. This work demonstrates the potential of SNEDDS as a versatile and biocompatible nanocarrier system capable of enhancing the therapeutic efficacy of paclitaxel while overcoming the solubility and toxicity challenges associated with conventional Cremophor EL–based formulations and provides a clearer relationship between the formulation and its biological effects compared to previous studies.

## 2. Materials and Methods

### 2.1. Materials

Paclitaxel was purchased from AA Blocks, Inc. (San Diego, CA, USA). Oleic acid, PEG 400, Tween 80, and 3‐(4,5‐dimethylthiazol‐2‐yl)‐2,5‐diphenyltetrazolium bromide (MTT) were obtained from Sigma‐Aldrich Co. Ltd. (St. Louis, MO, USA). Propidium iodide (PI) was supplied by Thermo Fisher Scientific (Waltham, MA, USA). All reagents were analytical grade and used as received.

### 2.2. Cell Lines and Authentication

Human cancer cell lines, including MCF‐7 (breast adenocarcinoma, hormone receptor‐positive; RRID: CVCL_0031), MDA‐MB‐231 (triple‐negative breast cancer; RRID: CVCL_0062), HeLa (cervical carcinoma; RRID: CVCL_0030), and HCT116 (colorectal carcinoma; RRID: CVCL_0291), were obtained from the Tissue Culture Unit, Department of Biochemistry, Faculty of Science, King Abdulaziz University (Jeddah, Saudi Arabia). All cell lines were originally purchased from the American Type Culture Collection (ATCC), Manassas, VA, USA.

Cell line identity was confirmed by short tandem repeat (STR) profiling, and morphological characteristics were verified through microscopic comparison with reference images. Routine mycoplasma screening was carried out using an enzyme‐linked immunosorbent (ELISA) assay, and all cultures were confirmed free of contamination before use in experiments.

All cell lines are widely used, human‐origin research resources with verified identity and contamination‐free status, supporting the reliability of the experimental outcomes.

### 2.3. Solubility Study

The solubility of paclitaxel was evaluated in aqueous medium as well as in the selected formulation components: oil (oleic acid), surfactant (Tween 80), and cosurfactant (PEG 400). An excess amount of paclitaxel was added to vials containing 3 mL of each vehicle. The mixtures were placed in a thermostatically controlled shaking water bath (Model 1031; GFL Corporation, Burgwedel, Germany) maintained at 25 ± 0.5°C for 72 h to reach equilibrium. Subsequently, the content of each vial was filtered using a Biomed Scientific sterile syringe filter (Nylon, 0.45 µm, hydrophilic, nonpyrogenic), and the filtrate was collected. The supernatants were appropriately diluted with ethanol, and the paclitaxel concentration was determined spectrophotometrically at 231 nm. Each experiment was performed in triplicate, and the results were expressed as mean ± standard deviation (mg/mL).

### 2.4. Preparation of SNEDDS Formulations

Oleic acid, Tween 80, and PEG 400 were chosen for SNEDDS development after solubility screening because they showed favorable paclitaxel solubilization compared with the aqueous medium. Therefore, they were considered suitable candidates for the oil, surfactant, and cosurfactant phases, respectively. Oleic acid was selected as the oil phase due to its good solvent capacity for paclitaxel and its biocompatibility and established suitability in lipid‐based drug delivery systems [20]. Tween 80 was selected as a nonionic surfactant because of its high drug solubilization potential, emulsification efficiency, and acceptable safety profile in pharmaceutical formulations [21]. PEG 400 was selected as the cosurfactant/cosolvent due to its ability to further enhance drug solubility, reduce interfacial tension, and improve the spontaneity of nanoemulsion formation [22]. In addition, all three excipients are widely available in pharmaceutically accepted grades and have been extensively used in SNEDDS and related lipid‐based formulations [23], making them practical and translationally relevant choices for the present study.

Four different SNEDDS formulations were prepared containing 10%–20% oil, 10%–80% PEG 400, and 10%–80% Tween 80. The specified quantities of each component (oil, surfactant, and cosurfactant) were accurately weighed and placed into screw‐cap vials. The mixtures were then vortexed until a homogeneous mixture was obtained. The combined proportion of the three components in each formulation totaled 100% (w/w). The detailed compositions of the prepared SNEDDS formulations are presented in Table 1.

**Table 1:** Composition and characterization of the prepared paclitaxel‐loaded SNEDDS formulation.

Run	Composition			Characterization		
	Oil (w/w)	PEG 400 (w/w)	Tween 80 (w/w)	Solubility (mg/mL)	Size (nm)	PDI
SNEDDS 1	10	80	10	7.17 ± 0.06	365 ± 38.74	0.76 ± 0.01
SNEDDS 2	10	45	45	10.74 ± 0.23	331 ± 19.86	0.55 ± 0.06
SNEDDS 3	10	10	80	18.86 ± 0.29	304.8 ± 11.96	0.49 ± 0.09
SNEDDS 4	20	40	40	13.65 ± 0.45	557.93 ± 48.95	0.71 ± 0.07

### 2.5. Characterization of the Prepared SNEDDS Formulations

The prepared paclitaxel‐loaded SNEDDS formulations were characterized for particle size, polydispersity index (PDI), and zeta potential using a Malvern Zetasizer Nano ZSP (Malvern Panalytical Ltd., Malvern, UK). Before measurement, each formulation was diluted tenfold with distilled water, and analyses were conducted at room temperature (25°C). Instrument parameters, including the number of runs, scans, voltage, and attenuation settings, were automatically optimized by the system. Each sample was analyzed in triplicate, and the mean values were reported.

The solubility of paclitaxel in the prepared SNEDDS formulations was determined by adding an excess amount of the drug to 3 mL of each formulation in screw‐capped glass vials. The mixtures were vortexed (Velp Scientifica ZX3) and maintained in a thermostatically controlled shaking water bath (GFL Corporation, Type 1083, Burgwedel, Germany) at 25 ± 0.5°C for 72 h to reach equilibrium. For drug quantification, paclitaxel was extracted from the SNEDDS by dilution with ethanol, which served as both a solvent and an emulsion‐disrupting agent. The samples were vortexed thoroughly to ensure complete disruption of the nanoemulsion system and full release of the incorporated drug, followed by filtration through a 0.45 µm syringe filter to remove any undissolved residues. The filtrate was appropriately diluted and analyzed spectrophotometrically at 231 nm. All experiments were performed in triplicate, and the results are expressed as mean ± standard deviation (mg/mL). UV–visible spectrophotometry was employed for paclitaxel quantification as a rapid and efficient analytical method suitable for formulation screening. To minimize potential interference from formulation excipients, a blank SNEDDS formulation containing all excipients without drug was used as a reference during analysis. Under these conditions, the selected excipients showed negligible absorbance at 231 nm, enabling reliable quantification of the drug.

Based on the results obtained, SNEDDS formulation 3 exhibited optimal physicochemical characteristics and the highest paclitaxel solubility. Therefore, it was selected for further investigation and designated as formulation F1. Additionally, a nonmedicated SNEDDS formulation was prepared and used for comparative evaluation throughout the study.

### 2.6. Cytotoxicity Assay

Human cancer cell lines, including breast cancer models (MCF‐7, hormone receptor‐positive, and MDA‐MB‐231, triple‐negative), cervical cancer (HeLa), and colon cancer (HCT116), were used to evaluate the cytotoxic potential of the developed formulations. Cells were cultured in T75 flasks using Gibco DMEM supplemented with 10% fetal bovine serum (FBS) and 1% antibiotic solution, under standard conditions (37°C, 5% CO_2_, and 95% relative humidity). When cultures reached ~90% confluency, adherent cells were detached using 0.25% trypsin (4 mL, 5 min, and 37°C), centrifuged to obtain a cell pellet, and resuspended in fresh complete medium. Cell viability was assessed using 0.4% trypan blue staining, and viable cells were counted with a hemocytometer.

For the cytotoxicity evaluation, 10,000 cells per well were seeded in 96‐well plates containing 0.1 mL of complete medium and incubated for 24 h to allow cell attachment. The attached cells were then treated with varying concentrations of F1 (3.125–25 µg/mL), the F2 nonmedicated SNEDDS formulation, and commercial paclitaxel “Paxol” Injection (6.25–50 µg/mL), all prepared in complete DMEM. Treatments were performed in triplicate and incubated for 24, 48, and 72 h to assess time‐dependent effects.

Following treatment, 100 µL of 3‐(4,5‐dimethylthiazol‐2‐yl)‐2,5‐diphenyltetrazolium bromide (MTT) solution (0.5 mg/mL in serum‐free medium) was added to each well, and plates were incubated in the dark for 3 h. The resulting formazan crystals were solubilized with 100 µL of DMSO and incubated for 15 min. Absorbance was measured at 595 nm using a Bio‐Rad microplate reader (Japan). The IC_50_ values were calculated from the dose–response curves using GraphPad Prism [9, 24, 25].

### 2.7. Investigation of Cell Morphology

The morphological changes in the studied cancer cell lines (MCF‐7, MDA‐MB‐231, HeLa, and HCT116) were examined following treatment with the IC_50_ concentrations of F1, Paxol, and pure paclitaxel. After treatment and incubation for an additional 24 h, the cells were observed under an inverted microscope, Olympus CKX53 (Tokyo, Japan) at 20× magnification. Morphological alterations were recorded and compared with those of untreated control cells to qualitatively assess the cytotoxic effects of the tested formulations.

### 2.8. Flow Cytometry Analysis of the Cell Cycle

PI was used to stain cellular DNA for the assessment of DNA content and cell cycle distribution via flow cytometry. MCF‐7 cells (1 × 10^6^) were seeded in small culture flasks and incubated for 24 h. The culture medium was then replaced with fresh medium containing the IC_50_ concentrations of SNEDDS formulation F1, commercial paclitaxel (Paxol), and pure paclitaxel, followed by incubation for an additional 24 h in a CO_2_ incubator (37°C, 5% CO_2_, and 90% humidity).

After treatment, cells were harvested by trypsinization, washed twice with phosphate‐buffered saline (PBS), and centrifuged at 1500 rpm for 5 min. The resulting cell pellets were fixed in 1 mL of 70% ethanol and stored at −20°C for at least 20 min. Subsequently, 100 µL of the fixed cell suspension was stained with 300 µL of PI solution (50 µg/mL) and incubated in the dark for 1 h at room temperature. The stained cells were then analyzed for DNA content and cell cycle phase distribution using a BD FACSCanto II flow cytometer (BD Biosciences, USA) [26, 27].

### 2.9. Assessment of Apoptosis in Treated MCF‐7 Cells

Apoptosis was evaluated using the FITC Annexin V/PI Apoptosis Detection Kit (BD Pharmingen Inc., San Diego, CA, USA) according to the manufacturer’s protocol. MCF‐7 cells (2 × 10^5^) were seeded in 6‐well plates and incubated for 24 h under standard culture conditions (37°C, 5% CO_2_, and 95% humidity). The culture medium was then replaced with fresh medium containing the IC_50_ concentrations of SNEDDS formulation F1, commercial paclitaxel (Paxol), and pure paclitaxel, followed by incubation for an additional 24 h.

After treatment, cells were harvested by trypsinization, centrifuged, and washed twice with PBS. The resulting cell pellets were resuspended in 100 µL of binding buffer, to which 10 µL of FITC Annexin V and 10 µL of PI were added. The mixture was gently vortexed and incubated in the dark for 20 min at room temperature. Following incubation, 400 µL of binding buffer was added to each sample prior to analysis.

Samples were analyzed using a BD FACSCanto II flow cytometer (BD Biosciences, USA), with fluorescence detection channels set for FITC (Annexin V) and PI. Data acquisition and analysis were performed automatically using the BD FACSDiva software [28].

### 2.10. Evaluation of MMP in Treated MCF‐7

The MMP (ΔΨm) of treated MCF‐7 cells was assessed using JC‐1 dye (5,5^′^, 6,6^′^‐tetrachloro‐1,1^′^, 3,3^′^‐tetraethylbenzimidazolylcarbocyanine iodide). MCF‐7 cells (2 × 10^5^ cells per well) were seeded in 6‐well plates and incubated for 24 h at 37°C in a CO_2_ incubator under standard conditions. The medium was then replaced with fresh medium containing the IC_50_ concentrations of SNEDDS formulation F1, commercial paclitaxel (Paxol), and pure paclitaxel, followed by incubation for an additional 24 h.

After treatment, cells were detached using 0.5 mL of 0.25% trypsin‐EDTA solution per well for 5 min, after which an equal volume of complete medium was added to neutralize the trypsin. The cell suspensions were collected, centrifuged to obtain pellets, and washed twice with PBS. The resulting cell pellets were resuspended in 300 µL of PBS containing 3 µL of 200 µM JC‐1 dye, yielding a final JC‐1 concentration of 2 µM. The cells were incubated in the dark at 37°C for 20–30 min and then washed with PBS to remove excess dye.

Fluorescence was analyzed using a flow cytometer, where JC‐1 aggregates, indicative of intact MMP (ΔΨm), emitted red fluorescence (590 nm), while JC‐1 monomers, indicative of depolarized mitochondria, emitted green fluorescence (530 nm). The red/green fluorescence intensity ratio was calculated, with a decrease in this ratio indicating mitochondrial depolarization [29].

### 2.11. Nuclear Staining of Treated MCF‐7 Cells

The nuclear morphology of treated MCF‐7 cells was evaluated using 4^′^, 6‐diamidino‐2‐phenylindole (DAPI) staining. MCF‐7 cells were treated with the IC_50_ concentrations of SNEDDS formulation F1, Paxol, and pure paclitaxel and then seeded into 24‐well plates and incubated for 24 h at 37°C under standard CO_2_ conditions, as described in the MMP assay.

After incubation, the cells were washed three times with PBS and fixed with 4% paraformaldehyde for 15 min at room temperature. Permeabilization was performed using 0.25% Triton X‐100 for 10 min, followed by three additional PBS washes. Subsequently, cells were stained with DAPI solution (1 µg/mL) and incubated overnight at 4°C in the dark.

After staining, cells were washed gently with PBS and examined under a fluorescence microscope. Representative images were captured to qualitatively assess nuclear morphology, including chromatin condensation and nuclear fragmentation, indicative of apoptosis [18].

### 2.12. Biocompatibility and Hemocompatibility Assessment

The biocompatibility of the developed SNEDDS formulation was evaluated using normal noncancerous cell lines and human fibroblasts (HFF‐1), employing MTT assays. Cells were treated with equivalent concentrations of the drug‐loaded SNEDDS, blank SNEDDS, and control media to differentiate formulation‐related effects from drug‐induced cytotoxicity. The selectivity index (SI) was also calculated as the ratio of the IC_50_ value in normal HFF‐1 cells to that in cancer cells (SI = IC_50_ normal cells/IC_50_ cancer cells).

In addition, hemocompatibility of the developed SNEDDS formulation was evaluated using a hemolysis assay, following a previously reported method with slight modification [30]. Briefly, 2 mL of fresh blood was collected from a healthy volunteer into a lavender‐top tube containing tripotassium EDTA (3 mg) as an anticoagulant. The sample was gently mixed and centrifuged to separate plasma, which was discarded. The obtained red blood cells (RBCs) were washed three times with PBS and centrifuged, and the supernatant was removed after each wash. The washed RBCs were then resuspended in PBS to a final volume of 4 mL. Aliquots of 0.2 mL of the RBC suspension were transferred into Eppendorf tubes, followed by the addition of 50 µL of paclitaxel‐loaded SNEDDS (F1, 50 µg/mL), Paxol (250 µg/mL), pure paclitaxel (250 µg/mL), or PBS (negative control), resulting in final concentrations of 10 µg/mL for F1 and 50 µg/mL for Paxol and paclitaxel. A positive control was prepared using deionized water to induce complete hemolysis. The samples were incubated at 37°C for 30 min and then centrifuged to separate intact cells. The supernatants were carefully collected and transferred to a 96‐well plate, and absorbance was measured at 570 nm using an ELISA reader. The percentage of hemolysis was calculated based on the measured absorbance, expressed as optical density (OD), using the following equation:

Hemolysis %=OD sample − OD negative controlOD positive control − OD negative control× 100.

## 3. Results and Discussion

Paclitaxel exhibited an aqueous solubility of 0.019 ± 0.001 mg/mL, which aligns with its known lipophilic nature and extremely low water solubility (reported to be less than 0.01 mg/mL) [19]. This inherent hydrophobicity significantly limits its oral bioavailability and poses formulation challenges for parenteral administration. To address these limitations, the solubility of paclitaxel was systematically evaluated in various excipients representing the three essential components of a SNEDDS: oil, surfactant, and cosurfactant. These components were selected based on their biocompatibility, GRAS status, and wide utilization in commercial oral and injectable formulations [31].

**Figure 1:**
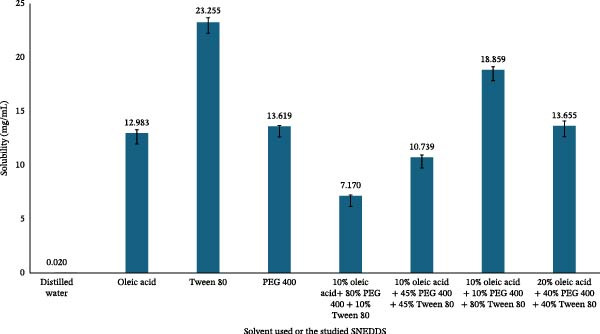
Solubility of paclitaxel in the studied solvents and SNEDDS formulations.

The solubility study revealed that paclitaxel exhibited markedly higher solubility in lipid‐based excipients compared to aqueous medium. Specifically, the solubility values were 12.983 ± 0.325 mg/mL in oleic acid, 23.255 ± 0.437 mg/mL in Tween 80, and 13.618 ± 0.082 mg/mL in PEG 400, as illustrated in Figure 1. These findings highlight the strong affinity of paclitaxel for surfactant and cosurfactant systems, suggesting that these excipients could effectively enhance the drug’s dissolution and facilitate its incorporation into SNEDDS formulations for improved solubilization and delivery.

Four paclitaxel‐loaded SNEDDS formulations were successfully prepared using varying ratios of oil, surfactant, and cosurfactant to optimize drug solubility and nanoemulsion characteristics. The selection of the component ranges for oil, surfactant, and cosurfactant was guided by our previously published work on drug‐loaded SNEDDS formulations [32]. That study demonstrated that formulations containing lower oil concentrations (10%–20%) produced smaller particle sizes, a finding attributed to the greater availability of surfactant molecules at reduced oil levels. These excess surfactant molecules effectively adsorb at the oil–water interface, forming a densely packed interfacial film that stabilizes the emulsion droplets. This tight packing reduces interfacial tension, minimizes droplet coalescence, and consequently leads to the formation of a stable nanoemulsion with a smaller globule size. Therefore, maintaining a lower oil concentration was essential to achieving the desired nanoscale characteristics and stability in the present formulation system.

All the prepared SNEDDS formulations exhibited clear to slightly opalescent appearances upon dilution, indicating efficient self‐emulsification. Characterization of the prepared SNEDDSs revealed noticeable differences in their physicochemical properties depending on the component ratios, as illustrated in Table 1. The prepared SNEDDS formulations exhibited almost neutral to slightly negative zeta potential values, which can be attributed to the combined effects of the carboxylic group of oleic acid, the neutral nature of paclitaxel, and the high proportion of nonionic surfactant (Tween 80) and cosurfactant (PEG 400) that effectively mask or neutralize surface charges at the droplet interface. Among the formulations, SNEDDS 3, composed of 10% oleic acid (oil), 10% PEG 400 (cosurfactant), and 80% Tween 80 (surfactant), demonstrated the highest paclitaxel solubility, suggesting enhanced drug incorporation capacity within the nanoemulsion system. Additionally, this formulation exhibited the smallest mean droplet size and the lowest PDI, indicating a uniform and stable nanoscale dispersion. These results suggest that the higher surfactant concentration in SNEDDS 3 improved interfacial stabilization and reduced droplet aggregation, leading to enhanced homogeneity and thermodynamic stability. Therefore, SNEDDS 3, designated as formulation F1, was selected as the optimized formulation for subsequent investigations, including cytotoxicity and mechanistic cellular assays.

### 3.1. Cytotoxicity Assay

**Figure 2:**
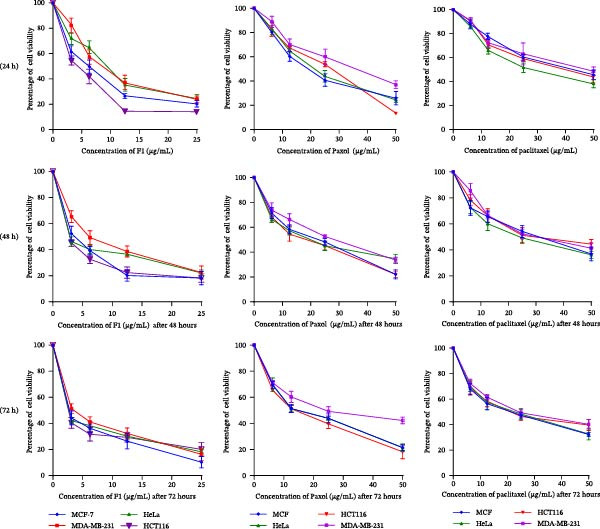
Cell viability (%) of MCF‐7, MDA‐MB‐231, HeLa, and HCT116 cancer cell lines following treatment with varying concentrations of paclitaxel‐loaded SNEDDS (F1), Paxol, and pure paclitaxel. Data are presented as mean ± SD (*n* = 3).

The cytotoxic effects of the tested formulations were evaluated in MCF‐7, MDA‐MB‐231, HeLa, and HCT116 cancer cell lines at 24, 48, and 72 h, as illustrated in Figure 2, with the corresponding IC_50_ values summarized in Table 2. A pronounced dose‐ and time‐dependent reduction in cell viability was observed for all treatments. At 24 h, the paclitaxel‐loaded SNEDDS formulation (F1) demonstrated enhanced cytotoxic activity compared with Paxol and pure paclitaxel, particularly in MCF‐7 and HCT116 cells.

**Table 2:** Cytotoxic activity (IC_50_ range and mean values) of the prepared formulations in the studied cancer cell lines.

Formulation	Conc. (µg/mL)	MCF‐7	MDA‐mb231	HeLa	HCT116	HFF‐1
F1 (24 h)	Range mean value	4.739–6.223	7.292–10.74	7.213 −10.26	3.046–4.429	—
		5.436 ± 0.742	8.848 ± 1.727	8.605 ± 1.525	3.687 ± 0.692	
F1 (48 h)	Range mean value	3.144–4.405	5.719 −7.692	3.118 −5.852	2.526–3.680	15.74–22.85
		3.733 ± 0.631	6.637 ± 0.987	4.309 ± 1.371	3.063 ± 0.577	18.96 ± 3.560
F1 (72 h)	Range mean value	2.530 −3.879	3.518–5.192	2.615–4.546	2.142–4.161	—
		3.152 ± 0.675	4.288 ± 0.838	3.479 ± 0.967	3.03 ± 1.012	
Paxol (24 h)	Range mean value	16.67–21.89	28.20–39.58	18.21–24.94	16.10–28.54	—
		19.1 ± 2.612	33.33 ± 5.699	21.3 ± 3.369	21.41 ± 6.242	
Paxol (48 h)	Range mean value	15.41–20.97	21.20 −28.34	14.99 −22.76	13.62 −18.63	61.48–86.93
		17.98 ± 2.767	24.49 ± 3.390	18.47 ± 6.840	15.94 ± 2.507	72.68 ± 12.755
Paxol (72 h)	Range mean value	1.122–1.238	1.269–1.476	1.119–1.242	1.066–1.180	—
		15.14 ± 2.036	23.52 ± 5.695	15.14 ± 2.152	13.29 ± 1.746	
Paclitaxel (24 h)	Range mean value	37.64–45.72	35.92–52.37	25.07–32.39	32.70–42.93	—
		41.44 ± 4.042	43.19 ± 8.243	28.47 ± 3.663	37.40 ± 5.121	
Paclitaxel (48 h)	Range mean value	20.99–31.21	25.72 −34.75	18.47–26.31	23.52–35.31	43.17–68.40
		25.56 ± 5.120	29.85 ± 4.520	22.02 ± 3.926	28.75 ± 5.907	53.92 ± 12.661
Paclitaxel (72 h)	Range mean value	15.45–22.43	19.37−28.66	16.28–23.75	16.12–26.60	—
		18.61 ± 3.495	23.53 ± 4.653	19.66 ± 3.741	20.68 ± 5.255	

With increasing incubation time (48 and 72 h), the cytotoxic effects became more prominent across all cell lines, as reflected by the progressive decrease in IC_50_ values (Table 2). Notably, F1 consistently exhibited the lowest IC_50_ values at all time points, confirming its superior anticancer efficacy. For example, in MCF‐7 cells, the IC_50_ value of F1 decreased from 5.436 ± 0.742 µg/mL at 24 h to 3.733 ± 0.631 µg/mL at 48 h and 3.152 ± 0.675 µg/mL at 72 h. In contrast, Paxol and pure paclitaxel showed higher IC_50_ values and a comparatively less pronounced reduction over time. Similar trends were observed in MDA‐MB‐231, HeLa, and HCT116 cells, indicating that F1 achieved both rapid onset and sustained cytotoxic effects.

Overall, these findings demonstrate that the developed SNEDDS formulation (F1) not only enhances the cytotoxic potency of paclitaxel but also improves its selectivity toward cancer cells over normal cells, supporting its potential as a safer and more effective alternative to conventional formulations. The improved performance of F1 is likely attributed to its nanoscale characteristics, which facilitate enhanced cellular uptake and sustained intracellular drug availability.

### 3.2. Cell Morphology Analysis

**Figure 3:**
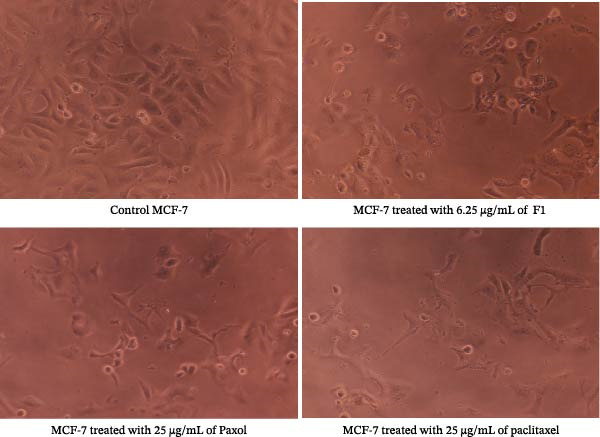
Representative microscopic images of MCF‐7 human breast cancer cells captured under an inverted phase‐contrast microscope (objective lens 20×, magnification 0.4 × 20) after 24 h of treatment with the IC_50_ concentrations of paclitaxel‐loaded SNEDDS (F1), Paxol, and pure paclitaxel.

**Figure 4:**
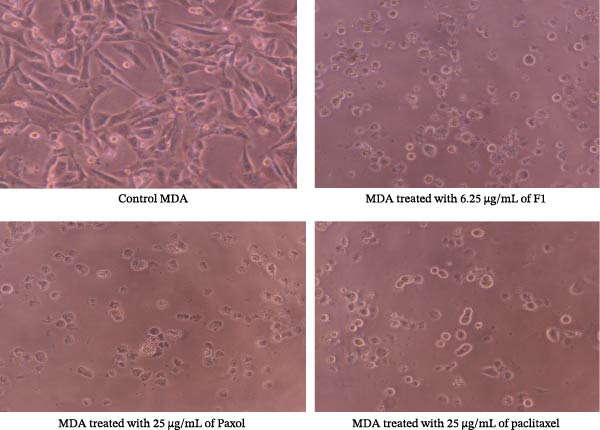
Representative microscopic images of MDA‐MB‐231 human triple‐negative breast cancer cells captured under an inverted phase‐contrast microscope (objective lens 20×, magnification 0.4 × 20) after treatment with the IC_50_ concentrations of paclitaxel‐loaded SNEDDS (F1), Paxol, and pure paclitaxel.

**Figure 5:**
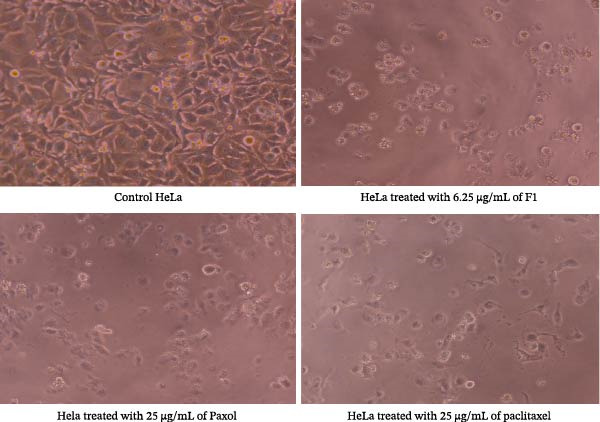
Representative microscopic images of HeLa human cervical cancer cells captured under an inverted phase‐contrast microscope (objective lens 20×, magnification 0.4 × 20) after treatment with the IC_50_ concentrations of paclitaxel‐loaded SNEDDS (F1), Paxol, and pure paclitaxel.

**Figure 6:**
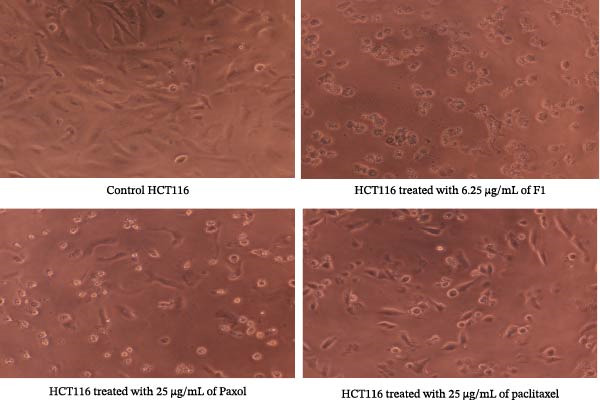
Representative microscopic images of HCT116 human colon cancer cells captured under an inverted phase‐contrast microscope (objective lens 20×, magnification 0.4 × 20) after treatment with the IC_50_ concentrations of paclitaxel‐loaded SNEDDS (F1), Paxol, and pure paclitaxel.

Microscopic examination of the treated cancer cell lines revealed distinct morphological alterations consistent with cytotoxic effects induced by the paclitaxel‐loaded SNEDDS formulation (F1). As illustrated in Figure 3 (MCF‐7), Figure 4 (MDA‐MB‐231), Figure 5 (HeLa), and Figure 6 (HCT116), cells exposed to F1 exhibited pronounced cell rounding, shrinkage, detachment, and disruption of monolayer integrity, indicating a significant loss of cellular viability. These morphological changes were markedly more evident in F1‐treated cells compared to those treated with pure paclitaxel or the commercial formulation (Paxol), which retained relatively more adherent and intact cells. In contrast, cells treated with the blank SNEDDS maintained normal spindle‐like morphology and confluence, confirming that the excipients alone did not contribute to cytotoxicity. The observed structural deterioration and detachment of cells following F1 treatment provide strong visual evidence supporting the enhanced cytotoxic potential and apoptotic activity of the optimized SNEDDS formulation across different cancer cell lines. These findings are in good agreement with previous studies reporting similar morphological alterations following treatment with hinokitiol phytosomal nanoformulation, confirming the characteristic cellular response to loss of membrane integrity [33].

MCF‐7 cells were selected for further cell cycle and apoptosis analyses due to their clinical relevance as a model for hormone receptor‐positive breast cancer, their well‐documented sensitivity to paclitaxel, and their suitability for mechanistic studies involving drug‐induced cytotoxicity and apoptotic pathways.

### 3.3. Analysis of the Cell Cycle

**Figure 7:**
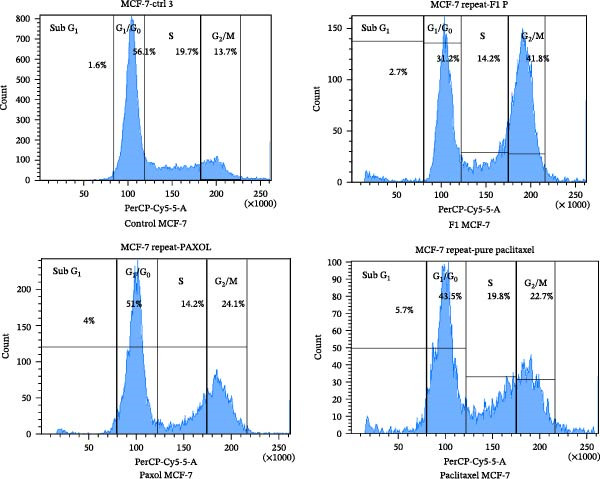
Flow cytometric analysis of cell cycle distribution in MCF‐7 cells treated with the IC_50_ concentrations of paclitaxel‐loaded SNEDDS (F1), Paxol, and pure paclitaxel.

**Figure 8:**
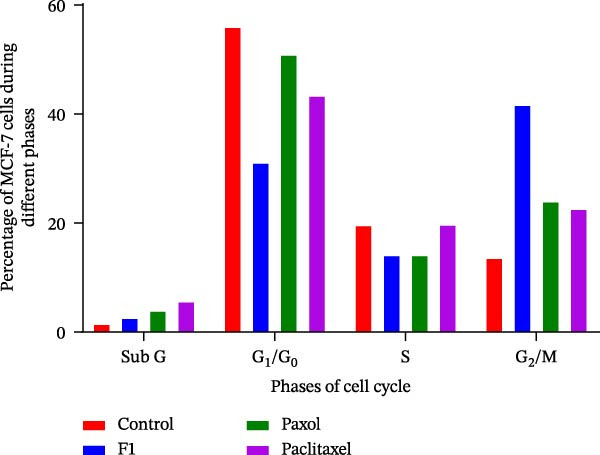
Quantitative representation of the percentage of MCF‐7 cells in different cell cycle phases (G_0_/G_1_, S, and G_2_/M) following treatment with the IC_50_ concentrations of paclitaxel‐loaded SNEDDS (F1), Paxol, and pure paclitaxel.

The cell cycle analysis of MCF‐7 cells treated with the IC_50_ concentrations of the formulated paclitaxel (F1), commercial paclitaxel (Paxol), and pure paclitaxel revealed significant alterations in cell cycle distribution compared to the untreated control (Table 3 and Figures 7 and 8). Paclitaxel exerts its anticancer activity primarily by inducing G_2_/M phase cell cycle arrest, which disrupts normal mitotic progression and ultimately leads to cell death. This effect arises from its ability to stabilize microtubules, preventing their depolymerization and thereby inhibiting the proper formation of the mitotic spindle [34]. As shown in Table 4, cells treated with the F1 SNEDDS formulation demonstrated a marked increase in the G_2_/M population (41.8%) compared with the control (13.7%), Paxol (24.1%), and pure paclitaxel (22.7%), indicating enhanced mitotic arrest. Concurrently, a notable reduction in the G_1_/G_0_ population (31.2%) was observed in F1‐treated cells, suggesting effective progression blockade at the G_2_/M checkpoint.

**Table 3:** Percentage of cells in MCF‐7 treated with IC50 of formulated paclitaxel (F1), Paxol, and paclitaxel during cell cycle phases.

Cell cycle phase (%)	Control	F1	Paxol	Paclitaxel
Sub G	1.6	2.7	4	5.7
G1/G_0_	56.1	31.2	51	43.5
S	19.7	14.2	14.2	19.8
G_2_/M	13.7	41.8	24.1	22.7

**Table 4:** The proportion of MCF‐7 cells exhibiting live, necrotic, early, and late apoptosis after being treated with F1, Paxol, and paclitaxel IC_50_ values.

Cell population distribution (%)	Control	F1	Paxol	Paclitaxel
Live	99.90%	26.20%	48.40%	53.30%
Necrosis	0%	3%	1.80%	1.40%
Early apoptosis	0.10%	64.40%	39.80%	32.80%
Late apoptosis	0%	6.20%	10%	12.40%
Total apoptosis	0.1	70.6	49.8	45.2

The enhanced G_2_/M accumulation observed with F1 treatment can be attributed to the improved solubility, cellular permeability, and intracellular delivery of paclitaxel achieved through the SNEDDS nanocarrier. The almost neutral surface charge and nanoscale globule size of F1 likely facilitated superior cellular uptake, allowing more efficient drug interaction with the microtubule network. This interaction disrupts spindle dynamics, leading to mitotic catastrophe, characterized by failed mitosis, multinucleation, and subsequent apoptosis or senescence. The mild increase in the sub‐G_1_ fraction (2.7%) in F1‐treated cells further supports early apoptotic activity. These findings are consistent with the known mechanism of paclitaxel‐induced microtubule stabilization, which activates cyclin B1/CDC2 complex accumulation and prevents the completion of mitosis [34].

Collectively, the data confirm that the F1 SNEDDS formulation significantly enhances paclitaxel’s ability to induce G_2_/M phase arrest, thereby amplifying its cytostatic and cytotoxic potential. This pronounced cell cycle arrest pattern supports the subsequent apoptosis results (Annexin V/PI assay), which further demonstrate that F1‐treated cells undergo a higher rate of programmed cell death compared to those treated with the free drug or commercial formulation.

### 3.4. Assessment of Apoptosis

**Figure 9:**
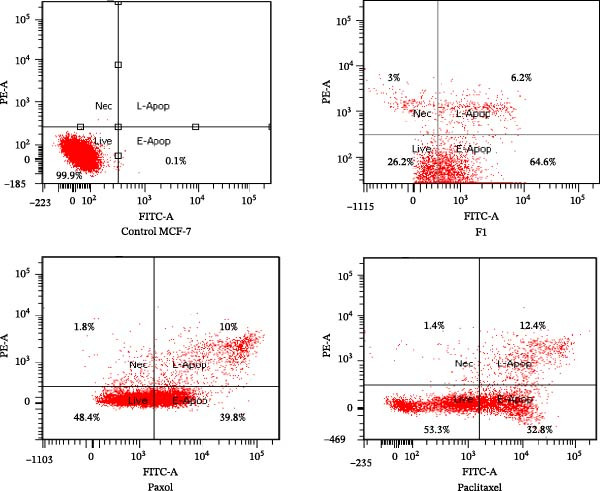
Flow cytometric analysis showing the proportion of live, necrotic, early apoptotic, and late apoptotic MCF‐7 cells treated with the IC_50_ concentrations of paclitaxel‐loaded SNEDDS (F1), Paxol, and pure paclitaxel.

Apoptosis was quantitatively assessed in MCF‐7 cells following treatment with the IC_50_ concentrations of paclitaxel‐loaded SNEDDS (F1), commercial paclitaxel (Paxol), and pure paclitaxel using the FITC Annexin V/PI assay (Table 4 and Figure 9). The data revealed that the F1 formulation induced the highest total apoptotic response (70.6%), predominantly in the early apoptotic phase (64.4%), compared to pure paclitaxel (49.8%) and Paxol (45.2%). The viable cell population markedly decreased to 26.2% following F1 treatment, whereas Paxol and pure paclitaxel retained 48.4% and 53.3% viability, respectively. Only a minor percentage of necrotic cells was observed across all treatments, confirming that the cell death process was primarily apoptotic rather than necrotic in nature.

The pronounced apoptotic effect observed with the F1 SNEDDS formulation can be attributed to its enhanced solubility, cellular uptake, and intracellular bioavailability of paclitaxel delivered via the nanoemulsion system. The nanosized droplets, stabilized by Tween 80 and PEG 400, likely facilitated greater membrane permeation and drug accumulation within cancer cells, leading to efficient engagement of microtubule targets and activation of apoptotic signaling pathways. Moreover, the substantial G_2_/M phase arrest (41.8%) induced by F1, as previously demonstrated in the cell cycle analysis, aligns with paclitaxel’s mechanism of action, wherein microtubule stabilization disrupts mitotic spindle function, resulting in mitotic catastrophe and subsequent apoptosis.

Interestingly, while F1 triggered a strong early apoptotic response, the progression to late apoptosis (6.2%) was relatively modest. This delayed transition may be explained by the presence of antiapoptotic proteins such as Bcl‐2 and partial activation of caspase cascades, which can transiently inhibit or delay full apoptotic execution. Previous studies have shown that caspase‐dependent apoptosis is often initiated following prolonged G_2_/M arrest, and resistance mechanisms inherent to MCF‐7 cells may further modulate the timing and extent of apoptotic progression [17].

Collectively, these findings indicate that the F1 SNEDDS formulation significantly enhances the proapoptotic efficacy of paclitaxel compared to both the pure drug and its commercial formulation, suggesting that nanocarrier‐mediated delivery not only improves drug bioavailability but also potentiates its mechanistic impact on cell cycle arrest and programmed cell death in breast cancer cells.

### 3.5. MMP

**Figure 10:**
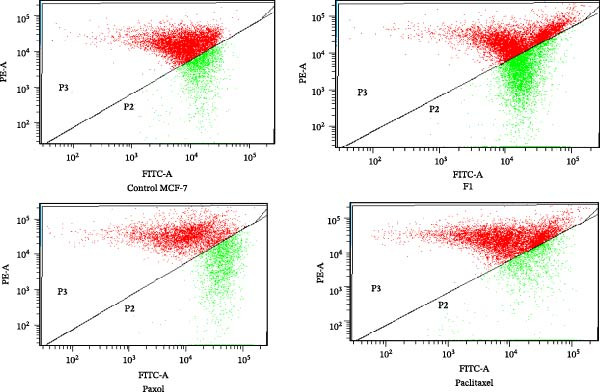
Flow cytometric analysis of mitochondrial membrane potential (MMP) in MCF‐7 cells following treatment with the IC_50_ concentrations of paclitaxel‐loaded SNEDDS (F1), Paxol, and pure paclitaxel.

The MMP serves as a key indicator of mitochondrial integrity and the initiation of intrinsic apoptotic signaling. The JC‐1 assay was employed to assess MMP disruption in MCF‐7 cells treated with IC_50_ concentrations of paclitaxel‐loaded SNEDDS (F1), Paxol, and pure paclitaxel (Table 5 and Figure 10). A marked reduction in JC‐1 dye aggregation reflects mitochondrial depolarization, indicative of early apoptotic signaling through cytochrome c release and caspase cascade activation [35].

**Table 5:** Percentage of mitochondrial membrane potential (MMP) via JC‐1 aggregation in MCF‐7 cells treated with F1, Paxol, and paclitaxel.

Treatment group	Percentage of JC‐1 aggregated	Ratio of aggregated JC‐1 to unaggregated
Control	75.3	3.04
F1	58.9	1.43
Paxol	46.3	0.46
Paclitaxel	59.5	1.47

The results demonstrated that Paxol induced the most pronounced MMP loss, with JC‐1 aggregation reduced to 46.3% (aggregation ratio 0.46), signifying substantial mitochondrial stress. However, this treatment produced comparatively lower total apoptosis (45.2%), suggesting that mitochondrial depolarization alone was insufficient to drive complete apoptotic execution, potentially due to limited drug uptake or suboptimal intracellular release kinetics. In contrast, both F1 and pure paclitaxel induced moderate MMP disruption (58.9% and 59.5%, respectively), accompanied by higher early apoptosis, particularly for F1 (64.4%).

The superior apoptotic efficiency of F1, despite its intermediate MMP loss, reflects a more effective initiation and propagation of mitochondrial‐mediated cell death, likely facilitated by improved drug solubilization, membrane permeability, and intracellular bioavailability provided by the SNEDDS system. This suggests that the extent of MMP loss does not directly correlate with apoptotic magnitude, emphasizing the importance of drug formulation and delivery kinetics in modulating apoptotic pathways.

Overall, the findings support the dual mechanism of paclitaxel action—microtubule stabilization leading to G_2_/M arrest and mitochondrial pathway activation leading to apoptosis [36]. The paclitaxel‐loaded SNEDDS formulation (F1) appears to optimize both mechanisms, enhancing therapeutic efficiency through improved mitochondrial engagement and apoptotic activation.

### 3.6. DAPI Staining of MCF‐7 Cells

**Figure 11:**
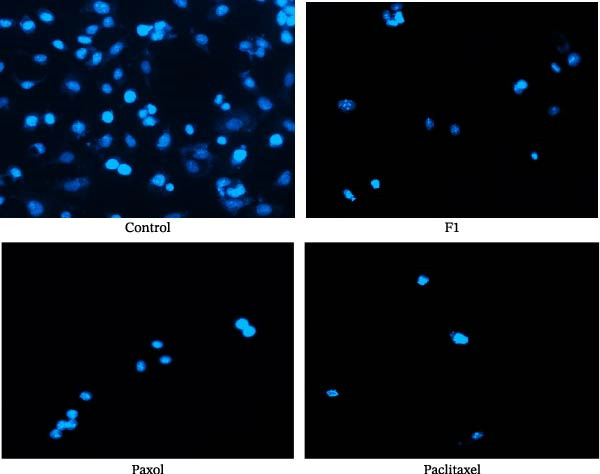
DAPI‐stained fluorescence microscopy images of MCF‐7 cells after treatment with the IC_50_ concentrations of paclitaxel‐loaded SNEDDS (F1), Paxol, and pure paclitaxel, showing nuclear condensation and fragmentation indicative of apoptosis.

To further confirm the cytotoxic and proapoptotic effects of the paclitaxel‐loaded SNEDDS formulation (F1) on MCF‐7 breast cancer cells, nuclear morphology was examined using DAPI staining under fluorescence microscopy. As illustrated in Figure 11, the nuclei of untreated control cells appeared intact, large, and uniformly stained, reflecting normal cellular morphology and viability. In contrast, cells treated with F1 exhibited a marked reduction in cell number and DAPI fluorescence intensity, accompanied by chromatin condensation and nuclear fragmentation, which are characteristic features of apoptosis. Treatment with pure paclitaxel similarly resulted in pronounced nuclear condensation and fragmentation, whereas Paxol‐treated cells showed reduced cell density and smaller nuclei compared to the control, indicating cytotoxic stress and apoptotic progression.

The greater degree of nuclear damage and morphological alterations observed in the F1‐treated group underscores the enhanced cytotoxic and apoptotic potency of the SNEDDS formulation compared to conventional paclitaxel and Paxol. These observations are consistent with the flow cytometry results demonstrating elevated early apoptosis (64.4%) and G_2_/M cell cycle arrest (41.8%), confirming that F1 promotes apoptosis through both mitotic disruption and nuclear disintegration pathways.

Although the promising paclitaxel‐loaded SNEDDS demonstrated enhanced cytotoxic and proapoptotic activity compared with pure paclitaxel and Paxol, cellular internalization was not directly evaluated. It was inferred from the observed biological effects. Additionally, while the blank SNEDDS showed minimal cytotoxicity, a comprehensive biocompatibility and toxicity assessment was beyond the scope of this study. Future work will include qualitative and quantitative cellular uptake studies using fluorescence‐based techniques, as well as detailed safety evaluations on normal cell lines and in vivo models to confirm internalization behavior and establish the formulation’s safety profile.

### 3.7. Biocompatibility and Hemocompatibility Assessment

To further evaluate formulation safety and selectivity, cytotoxicity was assessed in normal human fibroblast (HFF‐1) cells after 48 h of treatment. The results demonstrated that the paclitaxel‐loaded SNEDDS (F1) exhibited lower toxicity toward normal cells (IC_50_ = 18.96 ± 3.560 µg/mL) compared with its activity against cancer cell lines. Accordingly, the SI was calculated (Table 6), showing that F1 achieved the highest selectivity among all tested formulations, with SI values of 5.08 (MCF‐7), 2.86 (MDA‐MB‐231), 4.40 (HeLa), and 6.18 (HCT116). In contrast, Paxol demonstrated moderate selectivity, while pure paclitaxel exhibited the lowest SI values.

**Table 6:** Calculated selective index (SI) of MCF‐7, MDA‐mb231, HeLa, and HCT116 cancer cell lines after 48 h.

Formulation	MCF‐7	MDA‐mb231	HeLa	HCT116	Hemodialysis percentage
F1	5.08	2.86	4.4	6.18	1.1 ± 0.154
Paxol	4.04	2.96	3.95	4.56	0.8 ± 0.072
Paclitaxel	2.1	1.80	2.44	1.88	1.3 ± 0.143

In addition, hemocompatibility evaluation revealed that the hemolysis induced by F1, Paxol, and pure paclitaxel was below 2% (Table 6), indicating that all formulations exhibited negligible hemolytic activity and good compatibility with RBCs.

**Figure 12:**
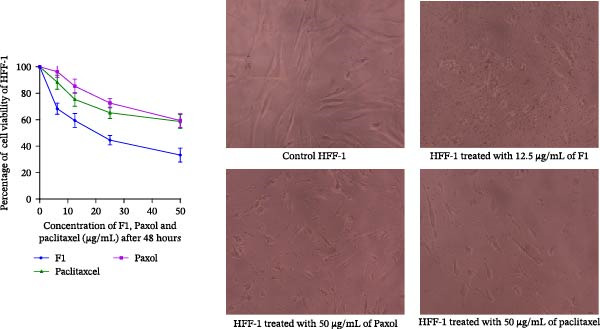
Cell viability (left panel) and microscopic images (right panel) of normal human fibroblast (HFF‐1) cells following treatment with IC_50_ concentrations of the studied formulations. Data for cell viability are presented as mean ± SD (*n* = 3).

The biocompatibility results (Figure 12) further support these findings, where the blank SNEDDS exhibited minimal cytotoxicity, maintaining high HFF‐1 cell viability across all tested concentrations, confirming the safety of the selected excipients. In comparison, the paclitaxel‐loaded SNEDDS (F1) showed a moderate, concentration‐dependent reduction in cell viability, which remained significantly lower than that observed in cancer cell lines. Pure paclitaxel exhibited comparatively higher cytotoxicity toward HFF‐1 cells than F1, indicating reduced selectivity. Overall, these results confirm that the developed SNEDDS formulation provides an improved safety and selectivity profile compared with conventional formulations.

## 4. Conclusion

Paclitaxel‐loaded SNEDDS was successfully developed using oleic acid, Tween 80, and PEG 400 to enhance the solubility and anticancer potential of paclitaxel. Among the prepared formulations, F1 (10% oleic acid, 10% PEG 400, and 80% Tween 80) exhibited the highest drug solubility, smallest particle size, and lowest PDI, with an almost neutral zeta potential contributing to formulation stability. In vitro studies using MCF‐7 breast cancer cells demonstrated that F1 markedly enhanced paclitaxel’s cytotoxicity compared to the pure drug and commercial Paxol. The formulation induced a pronounced G_2_/M cell cycle arrest (41.8%) and the highest total apoptosis (70.6%), predominantly in the early phase, supported by MMP loss and DAPI nuclear staining. These effects confirm that the nanoformulation facilitates enhanced cellular uptake and activation of mitochondrial‐mediated apoptotic pathways. All cell lines used in this study were authenticated and mycoplasma‐free, ensuring the validity and reproducibility of the biological findings. Overall, the developed SNEDDS represents a promising nanocarrier platform to improve the bioavailability, therapeutic efficacy, and safety profile of paclitaxel, offering potential clinical advantages in breast cancer therapy.

## Author Contributions

Conceptualization: Tarek A. Ahmed. Data curation: Tarek A. Ahmed, Ehab M. M. Ali, and Amjad Aljagthmi. Investigation: Tarek A. Ahmed, Ehab M. M. Ali, and Amjad Aljagthmi. Methodology: Tarek A. Ahmed, Ehab M. M. Ali, Abdulaziz A. Kalantan, and Amjad Aljagthmi. Resources: Tarek A. Ahmed. Software: Tarek A. Ahmed, Ehab M. M. Ali, and Amjad Aljagthmi. Validation: Tarek A. Ahmed, Ehab M. M. Ali, and Amjad Aljagthmi. Writing – original draft: Tarek A. Ahmed, Abrar Hakami, Amerh A. Alahmadi, Ehab M. M. Ali, and Amjad Aljagthmi. Writing – review and editing: Tarek A. Ahmed, Amerh A. Alahmadi, Alshaimaa M. Almehmady, and Abrar Hakami.

## Funding

This project was funded by the Deanship of Scientific Research (DSR) at King Abdulaziz University, Jeddah, Saudi Arabia (Grant IPP: 125‐166‐2025).

## Disclosure

All authors have read and agreed to the published version of the manuscript.

## Conflicts of Interest

The authors declare no conflicts of interest.

## Data Availability

The data generated in the present study are included in this article’s figures and/or tables.
